# Monomeric α-Synuclein Real-Time Induced Conversion: A New Approach to the Diagnostics of Neurodegenerative Synucleinopathies with Weak RT-QuIC Responses

**DOI:** 10.32607/actanaturae.27530

**Published:** 2025

**Authors:** D. A. Orlova, A. A. Kudriaeva, N. A. Kolotyeva, E. O. Ivanova, E. Yu. Fedotova, P. P. Tregub, A. B. Salmina, S. N. Illarioshkin, A. A. Belogurov Jr.

**Affiliations:** Shemyakin and Ovchinnikov Institute of Bioorganic Chemistry, Russian Academy of Sciences, Moscow, 117997 Russia; Brain Science Institute, Research Center of Neurology, Moscow, 125367 Russia; Department of Pathophysiology, Sechenov First Moscow State Medical University, Moscow, 119991 Russia; Department of Biological Chemistry, Russian University of Medicine, Moscow, 127473 Russia

**Keywords:** α-synuclein, synucleinopathies, multiple system atrophy, Lewy body dementia, real-time quakinginduced conversion (RT-QuIC), diagnostics

## Abstract

Neurodegenerative disorders classified as synucleinopathies (Parkinson’s disease, dementia with Lewy bodies, and multiple-system atrophy) are characterized by the accumulation of aberrant α-synuclein aggregates in neurons and glial cells. These diseases manifest clinically several years after the initial formation of pathological protein aggregates in the brain, making early and accurate diagnosis challenging. In recent years, a new method, which is based on real-time quaking-induced conversion (RT-QuIC) of α-synuclein, has been developed and validated. This technology holds great promise as a powerful diagnostic tool for the early and precise identification of synucleinopathies, potentially opening new horizons in the study of neurodegenerative diseases. RT-QuIC detects misfolded α-synuclein aggregates in human physiological fluids by introducing an excess of recombinant α-synuclein, which undergoes conformational conversion in an exponential, prion-like manner. The production of high-quality recombinant α-synuclein is a critical step in the effective application of this method, as protein purity significantly affects the sensitivity and specificity of the assay — key factors in its diagnostic utility. Using a three-step chromatographic purification protocol, we produced recombinant monomeric α-synuclein with a purity exceeding 97% from the periplasmic fraction of bacterial cells. While higher purity increases the assay duration, it also reduces the background signal and permits extended incubation times, which are essential for reliably detecting synucleinopathies with weak RT-QuIC responses, such as the cerebellar subtype of multiple-system atrophy. The data presented support the conclusion that optimized components of the RT-QuIC system will enable an accurate diagnosis of neurodegenerative synucleinopathies.

## INTRODUCTION

Synucleinopathies are a group of neurodegenerative diseases that include Parkinson’s disease, Lewy body dementia (LDB), and multiple system atrophy (MSA). The aggregation of a misfolded α-synuclein protein in neurons and/or glial cells plays a key role in the pathogenesis of these diseases: α-synuclein with an aberrant conformation has been found to be capable of trans-synaptic spreading throughout the central nervous system, like prions [1, 2, 3, 4]. α-Synuclein is a 14 kDa presynaptic protein encoded by the* SNCA *gene located on the long arm of chromosome 4 at locus 4q21–22. α-Synuclein is predominantly expressed in the midbrain substantia nigra, neocortex, and hippocampus [5]. Physiological α-synuclein levels are essential for normal mitochondrial functioning, neurotransmitter release, and maintenance of morphological cell integrity. Overexpression of α-synuclein and changes in its aggregation properties result in mitochondrial dysfunction, neuroinflammation, and impaired synaptic release of dopamine and other neurotransmitters, leading to neuronal death [6, 7]. A distinctive feature of the members of the synuclein family is their tendency to form aggregates. Native α-synuclein is an unstructured, monomeric soluble protein. In pathological conditions, it forms β-pleated oligomers (protofibrils) that are subsequently transformed into amyloid fibrils and deposited in neurons in the form of Lewy bodies and neurites, as well as other inclusions [8, 9, 10]. The mechanism of α-synuclein aggregate growth in each case is thought to be based on seed polymerization. Trans-synaptic spread of aberrant molecules from neuron to neuron is observed in Parkinson’s disease and LBD, whereas their accumulation and transmission in glial cells occurs in MSA [11].

To date, there has been no standardized reference method for detecting α-synuclein aggregates in the nervous system. Existing immunohistochemical approaches for identifying α-synuclein in peripheral tissue biopsies (e.g., skin, salivary glands, etc.) are technically complex and prohibitively expensive [12], which limits their routine use in clinical practice. Meanwhile, the development and implementation of highly sensitive techniques for detecting pathological forms of α-synuclein and other brain-derived proteins are critically important for improving diagnostic accuracy, particularly at the prodromal stage and for enabling timely therapeutic interventions in neurodegenerative diseases. One promising approach is the seed amplification assay (SAA), originally developed for the prion disease. This method exploits a protein misfolding chain reaction triggered by the presence of pathological protein conformers in patient-derived biological samples added to a reaction medium. Applying the prion hypothesis to α-synuclein in Parkinson’s disease, MSA, and DLB has spurred research into the potential of SAA to detect pathological α-synuclein conformers in various tissues and body fluids, including the skin, olfactory mucosa, cerebrospinal fluid, and the blood [12, 13].

A modern SAA version is the real-time quaking-induced conversion (RT-QuIC) assay [14, 15]. This assay uses a recombinant protein as a substrate, and the patient’s biological material serves as a “seed” to detect protein misfolding. RT-QuIC is based on the ability of a pathological α-synuclein form to induce conformational changes in normal monomeric α-synuclein, which leads to misfolded protein aggregation. The assay principle is to create artificial conditions for the seed amplification of α-synuclein by alternating the incubation and quaking cycles, which promotes additional fragmentation of the formed aggregates and an increase in protofibril formation. α-Synuclein aggregation is detected using a fluorescent dye, thioflavin T (ThT), that is incorporated into the aggregates during polymerization, which increases fluorescence over time [14, 16]. It should be noted that the purity of the substrate, monomeric soluble α-synuclein, is crucial for the reliability and reproducibility of results, as well as for the prevention of false-positive reactions [17, 18].

This study was aimed at improving the RT-QuICbased system for the diagnostics of neurodegenerative synucleinopathies and, in particular, at developing a method for the production of highly purified recombinant monomeric wild-type α-synuclein for its further use as a substrate in the RT-QuIC assay.

## EXPERIMENTAL

**Expression of recombinant α-synuclein in *Escherichia coli *cells**

The pET33b+ plasmid containing the human α-synuclein gene sequence was transformed into One Shot BL21 (DE3) Star *E. coli *cells (Thermo Fisher Scientific, USA). The cells were cultured in 500 mL of a bacterial LB medium containing 50 μg/mL kanamycin and 0.1% glucose under constant stirring at 200 rpm. The culture was grown to an optical density of 0.6 at a wavelength of 600 nm (OD_600_ = 0.6). Expression of the target protein was induced by adding isopropyl-β-D-l-thiogalactopyranoside (IPTG) to a final concentration of 1 mM, followed by incubation of the cells at 37°C and vigorous stirring for 4 h. Following the expression, the cells were pelleted by centrifugation at 4,000 *g *for 15 min.

**Periplasmic lysis**

After centrifugation, the cell pellet produced from 300 mL of the culture medium was resuspended in 60 mL of an osmotic shock buffer (30 mM Tris, 40% sucrose, and 2 mM EDTA, pH 7.2) and incubated at room temperature for 10 min. The suspension was centrifuged at 18,000 *g *for 20 min, the supernatant was separated, and the pellet was resuspended in 50 mL of ice water (dH_2_O) containing 20 μL of a saturated MgCl_2_ solution. The resulting suspension was kept on ice for 3 min, followed by centrifugation at 18,000 *g *for 20 min. The supernatant was dialyzed against a buffer containing 10 mM Tris and 1 mM EDTA (pH 7.2) at 4°C overnight.

**Ion exchange chromatography**

Ion exchange chromatography (IEC) was performed using a C 10/10 column (Cytiva, USA) packed with the Q Sepharose Fast Flow sorbent (Cytiva, USA) by means of a BioLab 30 fast protein liquid chromatography (FPLC) system (Jiangsu Hanbon Science and Technology Co., Ltd, China). Before loading the protein sample, the column was equilibrated with an IEC A buffer (10 mM Tris, pH 7.2). Prior to chromatography, all buffer solutions and protein samples were degassed and filtered through a 0.22 μm membrane filter. Elution was performed using a linear gradient of IEC A (10 mM Tris, pH 7.2) and IEC B (10 mM Tris and 0.15 M (NH_4_)_2_SO_4_, pH 7.2) buffers (7 column volumes), followed by a final column wash with a 100% IEC B buffer. The optical density of the eluate was monitored at a wavelength of 280 nm. To determine the time of α-synuclein desorption from the chromatographic column, the resulting fractions were collected and resolved on a 13% polyacrylamide gel electrophoresis (PAGE) under denaturing conditions, followed by staining with the Coomassie brilliant blue. Fractions containing protein bands corresponding to the molecular weight of monomeric α-synuclein were pooled and dialyzed against a 20 mM Tris buffer, pH 7.0 and 0.15(NH_4_)_2_SO_4_, overnight.

**Hydrophobic interaction chromatography**

Hydrophobic interaction chromatography (HIC) was performed using a C 10/10 column (Cytiva) packed with the Phenyl Sepharose High Performance sorbent (Cytiva). Before loading the protein sample, the column was equilibrated with HIC buffer A (50 mM bis-Tris and 1 M (NH_4_)_2_SO_4_, pH 7.0). The salt concentration in the samples was adjusted to 1 M by gradually adding (NH_4_)_2_SO_4_ while stirring the mixture at 4°C; the pH of the sample was adjusted to 7.0. Then, a sample was loaded onto the chromatographic column and eluted using a linear gradient of the HIC A (50 mM bis-Tris and 1 M (NH_4_)_2_SO_4_, pH 7.0) and HIC B (50 mM bis-Tris, pH 7.0) buffers (7 column volumes), followed by a final column wash with a 100% HIC B buffer. α-Synuclein-containing fractions were pooled and dialyzed against a 20 mM Tris buffer (pH 7.2) at 4°C overnight. The resulting protein solution was concentrated to 1.0–1.5 mg/mL using centrifugal concentrators with a cutoff of 5,000 Da and frozen at –80°C until further experiments.

**Gel-filtration chromatography**

Gel-filtration chromatography (GFC) was performed using a Superose 12 10/30 FPLC column (GE Pharmacia, USA). Before loading the protein, the column was equilibrated with a buffer (20 mM Tris, pH 7.2). Some 500 μ of a pre-concentrated sample was loaded onto the column at a flow rate of 1 mL/min. Chromatographic fractions were resolved on 13% PAGE under denaturing conditions, followed by staining with the Coomassie brilliant blue. The gel image was analyzed using the Image Lab Touch software for densitometric determination of protein purity. The resulting recombinant α-synuclein was aliquoted to the desired volume and stored at –0°C until the RT-QuIC assay.

**Sample collection and preparation**

To conduct pilot studies using the RT-QuIC technology, cerebrospinal fluid (CSF) samples (*n *= 3) were collected from patients with MSA (cerebellar type), LBD, and stiff-person syndrome (control), aged 58–69 years. Diagnoses were made based on anamnesis data, clinical examination, and the results of special laboratory and instrument tests, including high-field MRI (3 T) in the appropriate research modes. All patients gave written consent for the examination. The study was approved by the local ethics committee of the Research Center of Neurology (protocol No. 7-1/24). Lumbar puncture was performed in the morning, after overnight fasting. CSF was sterile-collected into a polypropylene tube, centrifuged, aliquoted to 500 μL portions, flash frozen, and stored at –80°C.

**RT-QuIC**

RT-QuIC assays were performed in black 96-well plates with an opaque bottom. Each well contained 37 ± 3 mg of glass beads (600–800 μm), a reaction buffer (100 mM phosphate buffer, pH 8.2, 10 μM ThT) containing recombinant α-synuclein at a final concentration of 0.1 mg/mL, and an undiluted CSF sample. The plate was sealed with an adhesive tape and placed in a ClarioStar multimodal plate reader (BMG Labtech). Samples were incubated at 37°C with intermittent shaking cycles for 125 h. Fibril formation kinetics were monitored by measuring the ThT fluorescence intensity at 450/480 nm every 60 min. Measurements were discontinued when the ThT fluorescence signal reached a plateau. Each sample was analyzed in triplicate.

## RESULTS

**Fig. 1:**
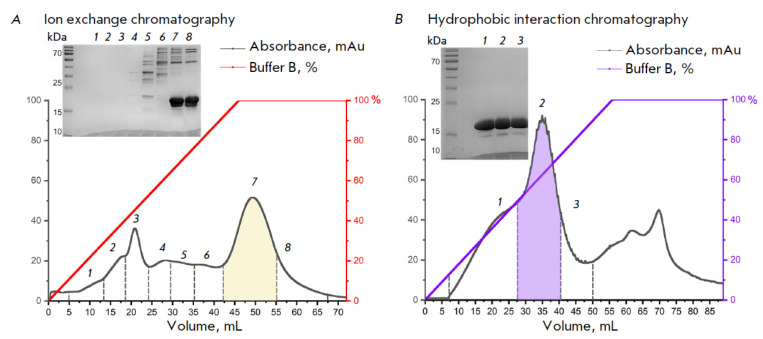
(A) Ion exchange chromatography of samples after periplasmic lysis. The peak highlighted in color corresponds to the fraction containing the largest amount of α-synuclein. The inset at the top left-hand side shows the electrophoresis of protein fractions after ion exchange chromatography. (B) Hydrophobic interaction chromatography of samples after ion exchange chromatography. The peak highlighted in color corresponds to the fraction containing the highest amount of α-synuclein. The inset at the top left-hand side shows the electrophoresis of protein fractions after hydrophobic interaction chromatography. The molecular weight of the target protein is 19 kDa (apparent molecular weight). The peak number on the chromatographic profiles corresponds to the PAGE lane number

The first step in the chromatographic purification of recombinant α-synuclein was ion exchange chromatography. The purity and separation efficiency of the protein fractions obtained during chromatography were assessed by electrophoretic analysis in a polyacrylamide gel in the presence of sodium dodecyl sulfate, followed by staining the gel with the Coomassie brilliant blue. Analysis of the fractions revealed the elution profile of α-synuclein from the chromatographic column. The main fraction containing α-synuclein is depicted in color on the chromatogram (*Fig. 1A*). According to electrophoretic analysis (*Fig. 1A*, inset), protein elution started at an IEC B buffer concentration of 60% in the eluent (with correction for the column volume). A further increase in the concentration of the IEC B buffer to 80% led to complete elution of α-synuclein. The α-Synuclein-containing fractions 7 and 8 were pooled and used in further steps in the protein purification.

α-Synuclein isolated from the periplasm contained protein impurities, so an additional purification step was required to prepare a homogeneous product. For this purpose, a hydrophobic chromatography step was introduced. Analysis of the chromatographic elution profile of α-synuclein showed that desorption of the target protein from hydrophobic sorbent started at an HIC B buffer concentration of 15% in the mobile was assessed using an electrophoretic analysis (*Fig. 1B*, inset). The PAGE results confirmed the removal of the major impurity proteins after the hydrophobic chromatography step. phase and continued up to 65%. The fraction containing the largest amount of α-synuclein is depicted in color on the chromatogram (*Fig. 1B*). The efficiency of protein purification by hydrophobic chromatography

**Fig. 2:**
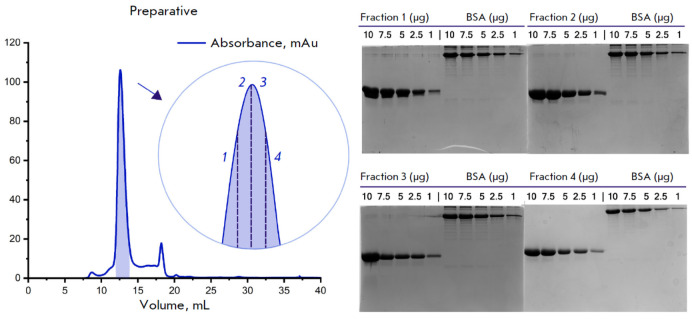
Size exclusion chromatography of α-synuclein. The peak highlighted in color corresponds to the fraction containing the target protein. At the top right-hand side, images of the denaturing electrophoresis of protein fractions are shown. α-Synuclein fractions at different concentrations (10, 7.5, 5, 2.5, and 1 μg) are presented on the left, and the molecular weight marker (BSA) at the same concentrations is presented on the right. The fraction numbers on the chromatogram correspond to those on PAGE images

Final purification of α-synuclein to separate possible covalent and non-covalent dimers was performed using size exclusion chromatography. The chromatographic elution profile of α-synuclein (*Fig. 2*) indicated that the protein retention time on the column was 12.5 min, which corresponded to its expected monomeric weight. Additional peaks did not contain a polypeptide component and corresponded to conductivity variations induced by the α-synuclein buffer components. Electrophoretic analysis of fractions 1–3 revealed additional upper bands, whereas fraction 4 was of the highest purity and was used for RT-QuIC.

**Fig. 3:**
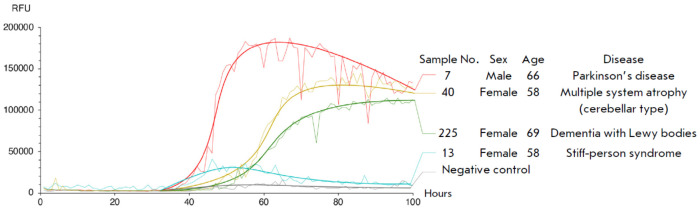
Curves of pathological α-synuclein amplification in patients (RFU – relative fluorescence units). Analysis of samples from patients with stiff-person syndrome (blue), Lewy body dementia (green), multiple system atrophy of the cerebellar type (yellow), and Parkinson’s disease (red)

The content of aberrant α-synuclein in patients’ CSF was measured using a modified RT-QuIC protocol [16]. During the measurement, pathological α-synuclein aggregates were partially denaturated by periodic quaking. With an excess of the recombinant monomeric protein in the reaction mixture, the misfolded aggregated protein, which binds to ThT, is amplified and enriched, which leads to an increase in the fluorescence intensity. The results are shown in *Figure 3*.

Sample 13 (stiff-person syndrome, SPS) had no significant increase in fluorescence, whereas samples 40 (multiple system atrophy, MSA) and 225 (Lewy body dementia, LBD) showed a fair increase in the fluorescence signal in the 40–60 h interval, which reached 145,000 and 120,000 RFU, respectively. Sample 7 (Parkinson’s disease) showed the highest fluorescence signal of 170,000 RFU and the highest rate at reaching the plateau in an interval of 32–45 h. Thus, the analysis of RT-QuIC curves revealed an increase in the fluorescence level to 120,000–150,000 RFU in CSF samples from MSA and LBD patients, respectively, after about 75 h of observation and 170,000 RFU from PD after 55 h of observation. There was no increase in fluorescence in the CSF sample from the SRS patient, indicating the lack of aberrant α-synuclein in the biomaterial. A cerebrospinal fluid analogue (100 mM NaCl, 2 mM KCl, 1 mM CaCl_2_, 5 mM urea, 300 μg/mL BSA, 2.5 mM glucose, NaHCO_3_, pH 7.3) was used as a negative control.

## DISCUSSION

The presented results indicate that the produced recombinant monomeric α-synuclein is the optimal substrate for use in the RT-QuIC technology. Previously, a variety of purification methods and extraction buffer compositions had shown to significantly affect the conformation, stability, and aggregation of α-synuclein, which, in turn, significantly complicates the interpretation of the assay results [17, 18]. In addition, standardization of methods for the production and purification of recombinant α-synuclein for diagnostic purposes is topical. By using the sequential combination of ion exchange and hydrophobic chromatography, we produced a highly purified target protein (≥97% purity) which corresponds to recombinant monomeric α-synuclein and that may be successfully used in further functional tests. Such high purity and homogeneity of α-synuclein are the key factors in maintaining the reproducibility of laboratory tests for synucleinopathies. In the future, this will ensure greater reliability of RT-QuIC data and facilitate the jump from laboratory results to clinical practice.

Diagnosis of synucleinopathies is a complex task that requires high accuracy. In this pilot study, we used the RT-QuIC assay to analyze samples from patients with moderate LBD and MSA of the cerebellar type (MSA–M) who had impaired cognitive status. Samples derived from patients with stiff-person syndrome were used as a negative control. It should be noted that MSA can be diagnosed with 100% accuracy only postmortem, because its clinical picture overlaps with that of other synucleinopathies. There are two main subtypes, parkinsonian (MSA–P) and cerebellar (MSA–C), of this rare and rapidly progressing neurodegenerative disease. Clinical presentation of MSA–P includes symptoms typical of classical parkinsonism, while MSA–C is characterized by cerebellar ataxia. Similarly, the diagnosis of LBD is complicated by overlapping symptoms. LBD is often misdiagnosed as Alzheimer’s disease due to the similarity of clinical manifestations. In this case, the only reliable diagnostic marker is the detection of aberrant α-synuclein in the patient.

The results of this study indicate that the onset of significant synuclein aggregation begins 50 h after the mixing of CSF samples with reaction mixture components. But in similar studies, the rise of aggregation curves started after approximately 12 h of incubation [19]. The rate of α-synuclein aggregation can depend on many factors, and the degree of purification is obviously the most critical one. Oligomers that could not be eliminated during recombinant protein purification can induce aggregation in the same way as natural pathological variants introduced into the reaction mixture do. On the one hand, a higher degree of α-synuclein purification increases the assay duration; on the other hand, it significantly reduces the background noise and allows for extended incubation times, which are essential for the reliable detection of synucleinopathies such as MSA, which are known for their weak seeding activity in the RT-QuIC assay [19].

## CONCLUSIONS

Early detection of the aberrant proteins that are involved in neurotoxicity/neuroinflammation mechanisms and present in the systemic bloodstream and CSF is currently considered as one of the most critical frontiers in neuroscience research [20]. A series of pilot studies to quantify pathological aggregates in CSF from patients with synucleinopathies using RT-QuIC and highly purified recombinant α-synuclein have demonstrated the significant potential of this approach in advancing laboratory-based neurodiagnostics. Further research will need to be focused on the development of a standardized method for detecting pathological forms of α-synuclein that is informative, highly sensitive, specific, reproducible, and user-friendly, ensuring its suitability for future implementation in clinical practice.

## Abbreviations

CSF: cerebrospinal fluid
LBD: Lewy body dementia
EDTA: ethylenediaminetetraacetic acid
FPLC: fast protein liquid chromatography
SEC: size exclusion chromatography
HIC: hydrophobic interaction chromatography
IEC: ion exchange chromatography
IPTG: isopropyl-β-D-1- thiogalactopyranoside
MRI: magnetic resonance imaging
MSA-C: multiple system atrophy of the cerebellar type
MSA: multiple system atrophy
MSA-P: multiple system atrophy of the parkinsonian type
PD: Parkinson’s disease
PAGE: polyacrylamide gel electrophoresis
RT-QuIC: real-time quaking-induced conversion
SAA: seed amplification assay
SPS: stiff-person syndrome
ThT: thioflavin T
